# The *Escherichia coli* uhp two-component system functions as a sensory channel for hexose phosphates secreted by *Bifidobacterium*

**DOI:** 10.1038/s41598-026-64276-3

**Published:** 2026-08-25

**Authors:** Tomotsugu Ohno, Masato Hoshino, Tsubasa Niino, Masanori Tamura, Miho Yoshimura, Kaneyoshi Yamamoto

**Affiliations:** Department of Frontier Bioscience and Research Institute of Micro-Nano Technology, Hosei University, Koganei, Tokyo, 184-8584 Japan

**Keywords:** *Escherichia coli*, *Bifidobacterium*, Uhp two-component system, Glucose-6-phosphate, Inter-species signaling, Kinetic trap, Biochemistry, Microbiology

## Abstract

The human gut microbiota relies on inter-species communication for stability, yet interactions between non-dominant taxa like *Pseudomonadota* (*Escherichia coli*) and *Actinomycetota* (*Bifidobacterium*) remain poorly understood. We demonstrate that *E. coli* repurposes its Uhp two-component system as a specialized sensory channel to monitor *Bifidobacterium* metabolic activity. Screening 54 transport systems revealed that *uhpT* and *idnT* genes are specifically induced by *Bifidobacterium* supernatants, a response absent in members of the *Bacteroidota*. Using an enzymatic “kinetic trap” approach with a slow-acting Zwf mutant (D177N), we provide biochemical evidence that glucose-6-phosphate (G6P) is the primary signaling molecule. Experimental validation using Modified Garche’s Medium confirms that G6P secretion is a consistent byproduct of bifidobacterial metabolism occurring during growth on diverse human milk oligosaccharide (HMO) constituents, including galactose. Furthermore, *idnT* activation occurs independently of Uhp-mediated transport, suggesting the secretion of a multifaceted metabolic cocktail. We propose these molecular links form a “non-dominant network” enabling low-abundance species to coordinate metabolism and maintain stability alongside the dominant majority. These findings redefine the physiological role of the Uhp system and offer a new perspective on the regulatory networks governing the human microbiome. (183 words)

**Supplementary Information:**

The online version contains supplementary material available at 10.1038/s41598-026-64276-3.

## Introduction

**Fig. 1:**
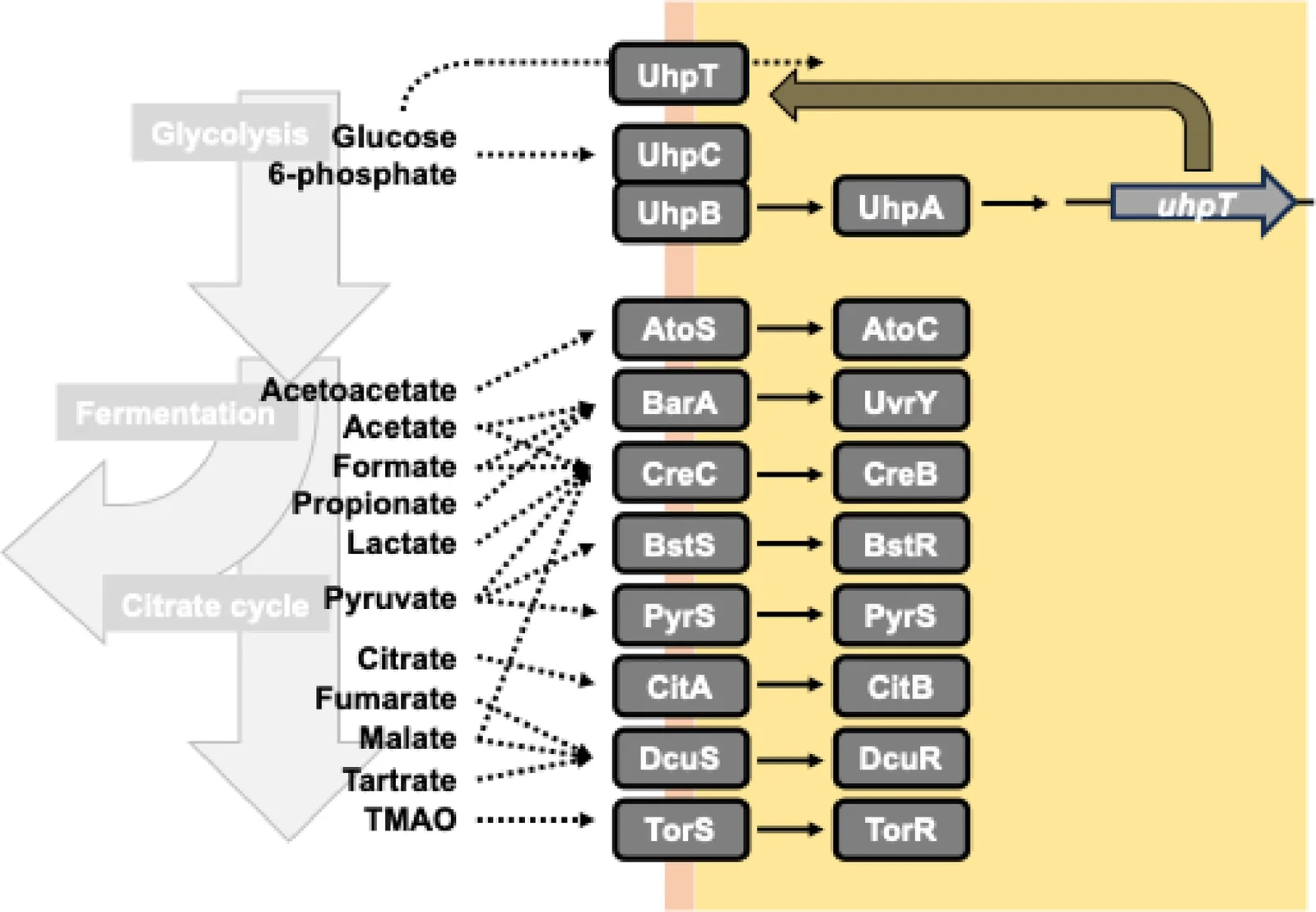
**Representative metabolic-sensing two-component systems in** ***Escherichia coli***. A schematic overview illustrates the relationship between primary biological metabolites and their corresponding two-component systems (TCSs). (Left) Major intermediates derived from glycolysis, fermentation, and the citrate cycle serve as environmental stimuli. Dashed arrows indicate the perception of specific ligands by their respective membrane-bound sensor histidine kinases (HKs). (Right) Signal transduction occurs from the HKs to their cognate response regulators (RRs) (solid arrows) to regulate downstream genetic responses. Among these, the Uhp two-component system is uniquely specialized for the sensing of extracellular glucose 6-phosphate (G6P). The signaling cascade is initiated via the membrane-bound receptor UhpC, which triggers the UhpB-UhpA phosphorelay to induce the expression of the hexose-6-phosphate transporter (*uhpT*).

Bacteria utilize two-component systems (TCSs) to sense and adapt to diverse environmental stimuli, with several systems in *Escherichia coli* specialized for monitoring primary biological metabolites^1^. Notable examples of such metabolic sensors include AtoS-AtoC for acetoacetate^2^, CitA-CitB for citrate^3^, and DcuS-DcuR for C4-dicarboxylates^4^(Fig. 1). The Uhp system is uniquely dedicated to the sensing and transport of hexose-6-phosphates, particularly glucose-6-phosphate (G6P), a key intermediate in glycolysis^5,6^(Fig. 1). The induction of the *uhpT* transporter gene is tightly controlled by the Uhp two-component system^7^. This process is initiated when extracellular G6P binds to the membrane-bound receptor UhpC, which subsequently activates the sensor histidine kinase UhpB and the response regulator UhpA to initiate transcription^8–10^. The Uhp system exhibits exquisite ligand specificity; induction strictly requires a phosphate group at the C6 position and a hydroxyl group at the C1 position of the glucose backbone, making the system highly sensitive to the structural integrity of the inducer^11,12^.

While the molecular mechanisms of the Uhp system have been well-characterized in isolation, its physiological function within the complex microbial community of the gut remains underexplored. Current understanding of gut microbiota dynamics often focuses on the competitive relationships between dominant phyla, such as *Bacteroidota* and *Bacillota*^13,14^. However, non-dominant phyla, including *Pseudomonadota* (e.g., *Escherichia coli*) and *Actinomycetota* (e.g., *Bifidobacterium*), may engage in more nuanced molecular interactions^15–17^. Certain species, most notably *Bifidobacterium bifidum*, are known for their specialized ability to degrade human milk oligosaccharides (HMOs) using extracellular glycosidases, thereby releasing metabolic byproducts into the surrounding environment^18–21^.

In this study, we demonstrate that the *Escherichia coli* Uhp system serves as a specialized sensory channel for detecting hexose phosphates secreted by *Bifidobacterium*. By integrating phenotype microarray analysis with enzymatic “kinetic trap” approach, we provide evidence suggesting that this inter-species signaling mechanism allows *Escherichia coli* to monitor the metabolic activity of *Bifidobacterium*, revealing a novel layer of molecular communication among non-dominant members of the gut microbiota.

## Results

### Identification of *Escherichia coli* Transporters Induced by *Bifidobacterium* Secretions

**Fig. 2:**
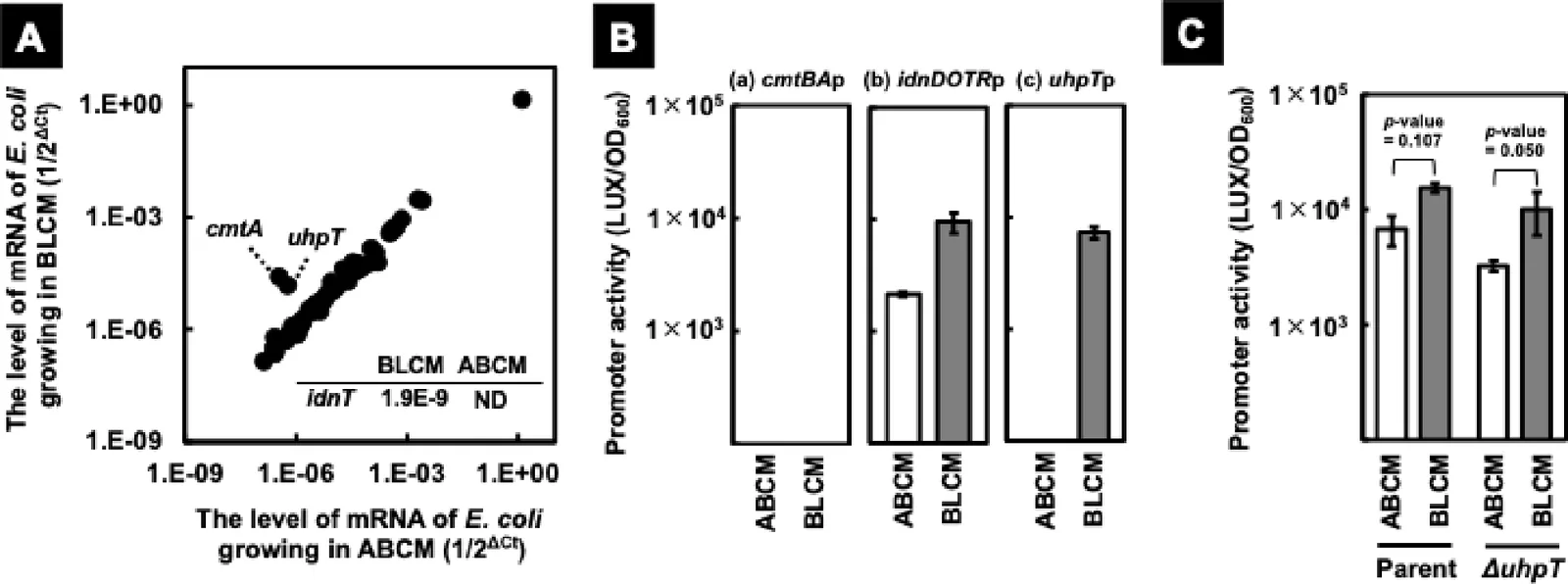
**Transcriptional induction of** ***uhpT*** **in response to metabolic secretions from** ***Bifidobacterium***. [A] Scatter plot analysis of relative mRNA levels. *Escherichia coli* W3110 was cultured for 2.5 h in control ABCM medium or Bifidobacterium-conditioned medium (BLCM; ABCM supplemented with Bifidobacterium longum subsp. longum JCM 1217 supernatant). Transcript levels of 54 representative transporter genes were determined by RT-qPCR. Candidate genes exhibiting significant induction (*cmtA*, *idnT*, and *uhpT*) are highlighted. [B] Promoter activities of *cmtBA*,* idnDOTR*,* uhpT* in W3110 typeA, measured by the luciferase reporter system (pLUX-cmtBA, pLUX-idnDOTR, and pLUX-uhpT). Normalized bioluminescence (LUX/OD_600_) was monitored after 2.5 h of growth in the indicated media. Data represent the means ± SD from three independent experiments. [C] Promoter activity of *idnDOTR* in BW25113 (Parent) and JW3641ΔKm (*ΔuhpT*) cultured in ABCM and BLCM. Normalized bioluminescence (LUX/OD_600_) was monitored after 2.5 h of growth in the indicated media. Data represent the means ± SD from three independent experiments. Statistical significance between ABCM and BLCM conditions was evaluated using a two-tailed Student’s t-test (*p*-values are indicated above the brackets).

To identify the specific transporters involved in the interaction between *Escherichia coli* and *Bifidobacterium*, we initially screened 54 representative transport systems corresponding to the 61 carbon sources utilized by *Escherichia coli* W3110 (Table S1). RT-qPCR analysis revealed that three genes—*cmtA* (mannitol-specific PTS), *idnT* (gluconate transporter), and *uhpT* (hexose-6-phosphate transporter)—exhibited significant induction in the presence of *Bifidobacterium longum* subsp. *longum* JCM 1217-conditioned medium (BLCM) (Fig. 2A). Subsequent luciferase reporter assays were performed to determine whether this induction occurred at the transcriptional level. The *cmtA* and *idnT* genes are co-transcribed as part of the *cmtBA* and *idnDOTR* operons, respectively^22^, whereas *uhpT* undergoes monocistronic transcription^10^. The results showed that the promoters of *idnDOTR* and *uhpT* were significantly activated by BLCM (14-fold and detectable induction, respectively), whereas the *cmtBA* promoter remained inactive (Fig. 2B). This suggests that the observed increase in *cmtA* mRNA may involve post-transcriptional mechanisms. Critically, we investigated whether the co-induction of *uhpT* and *idnT* followed a sequential model, where G6P imported via UhpT is metabolized to intracellular gluconate to trigger *idnT*^23–25^. Our experiments using a Δ*uhpT* mutant (JW3641ΔKm) demonstrated that the induction of the *idnDOTR* promoter remained fully intact even in the absence of hexose phosphate transport (Fig. 2C). This finding disproves the sequential induction hypothesis and indicates that the *Bifidobacterium* supernatant contains multiple distinct inducing factors—likely both hexose phosphates and gluconate (or its analogs)—that are sensed by *Escherichia coli* through independent regulatory pathways. These results suggest that *Escherichia coli* utilizes its Uhp and Idn systems to monitor a complex cocktail of metabolic footprints secreted by *Bifidobacterium*.

### Regulatory Requirements for *uhpT* Induction by *Bifidobacterium* Secretions

**Fig. 3:**
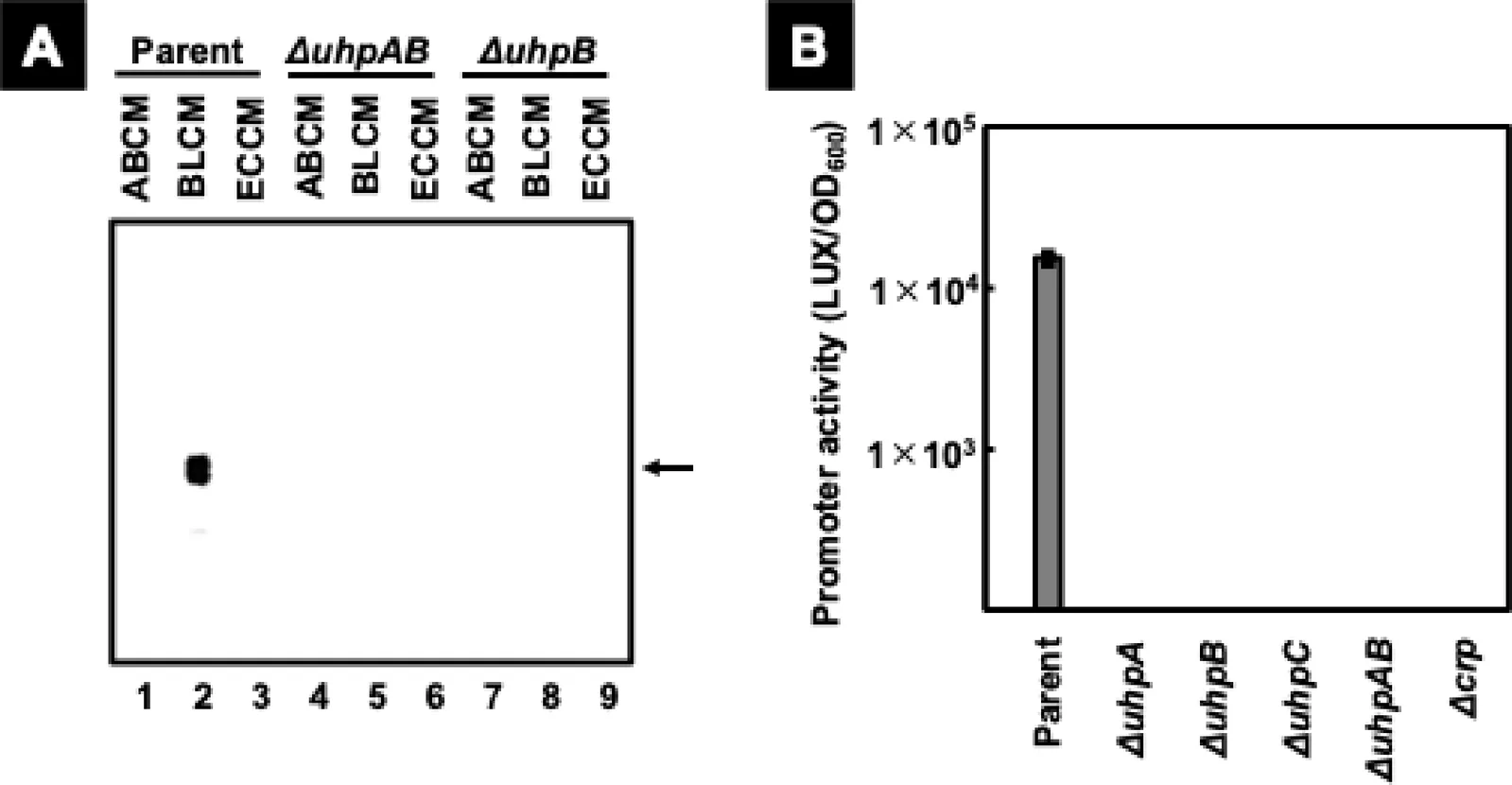
**Dependency of** ***uhpT*** **induction on the Uhp two-component system and cAMP receptor protein (CRP).** [A] Northern blot analysis of *uhpT* mRNA. Total RNA was isolated from *Escherichia coli* cells: BW25113 (Parent) (lanes 1–3), BW27871 (*ΔuhpAB*) (lanes 4 to 6), JW3643ΔKm (*ΔuhpB*). (lanes 7–9) Cells were grown in ABCM (control) (lanes 1, 4, and 7), *Bifidobacterium*-conditioned medium (BLCM; *Bifidobacterium longum* subsp. *longum* JCM 1217 supernatant) (lanes 2, 5, and 8), or *E. coli*-conditioned medium (ECCM) (lanes 3, 6, and 9). A DIG-labeled probe specific to the *uhpT* coding region was used for detection. The membrane image was cropped and adjusted for contrast using ImageJ. The full-length, uncropped chemiluminescent image of the Northern blot is provided in Figure S1 in the Supplementary Information. [B] Promoter activity of *uhpT* in BW25113 (Parent), JW3644ΔKm (*ΔuhpA*), JW3643ΔKm (*ΔuhpB*), JW3642ΔKm (*ΔuhpC*), BW27871 (*ΔuhpAB*), and JW5702ΔKm (*Δcrp*) cultured in BLCM medium. Normalized bioluminescence (LUX/OD_600_) was monitored after 2.5 h of growth in the indicated media. Data represent the means ± SD from three independent experiments. Statistical significance between the Parent and each mutant was evaluated using a two-tailed Student’s t-test (*p* < 0.001 for all mutants compared to the Parent in BLCM).

Transcriptional activation of *uhpT* specifically requires the Uhp two-component system. To verify that the *uhpT* induction observed in the initial screen occurred at the transcriptional level, we performed Northern blot analysis. A distinct transcript corresponding to *uhpT* was detected exclusively in cells cultured in *Bifidobacterium longum* subsp. *longum* JCM 1217-conditioned medium (BLCM), whereas no signal was observed in the control medium (ABCM) or *Escherichia coli* conditioned medium (ECCM) (Fig. 3A). This induction was completely abolished in Δ*uhpAB* and Δ*uhpB* mutant backgrounds, confirming that the response is not a generalized metabolic shift but a specific signaling event mediated by the Uhp system (Fig. 3A). The UhpABC receptor-histidine kinase complex and the global regulator cAMP receptor protein (CRP) are indispensable for the response^8–10^. The genetic requirements for *uhpT* activation were further characterized using a luciferase reporter assay in various deletion mutants. In the presence of BLCM, the significant promoter activity observed in the wild-type strain was entirely eliminated by the individual deletion of the response regulator (*uhpA*), the sensor kinase (*uhpB*), or the membrane-bound receptor (*uhpC*) (Fig. 3B). These results demonstrate that *Escherichia coli* perceives the *Bifidobacterium*-derived signal through the canonical UhpABC sensory cascade, where UhpC-mediated detection of an external ligand triggers the UhpB-UhpA phosphorelay. Additionally, we found that *uhpT* promoter activity was lost in the Δ*crp* strain, confirming that the global regulator cAMP receptor protein (CRP) is essential for transcriptional initiation at this locus (Fig. 3B). Collectively, these data establish that *Escherichia coli* utilizes the high-affinity Uhp two-component system to specifically monitor metabolic secretions from *Bifidobacterium*.

### Genus-Specific Induction of the *uhpT* Promoter

**Fig. 4:**
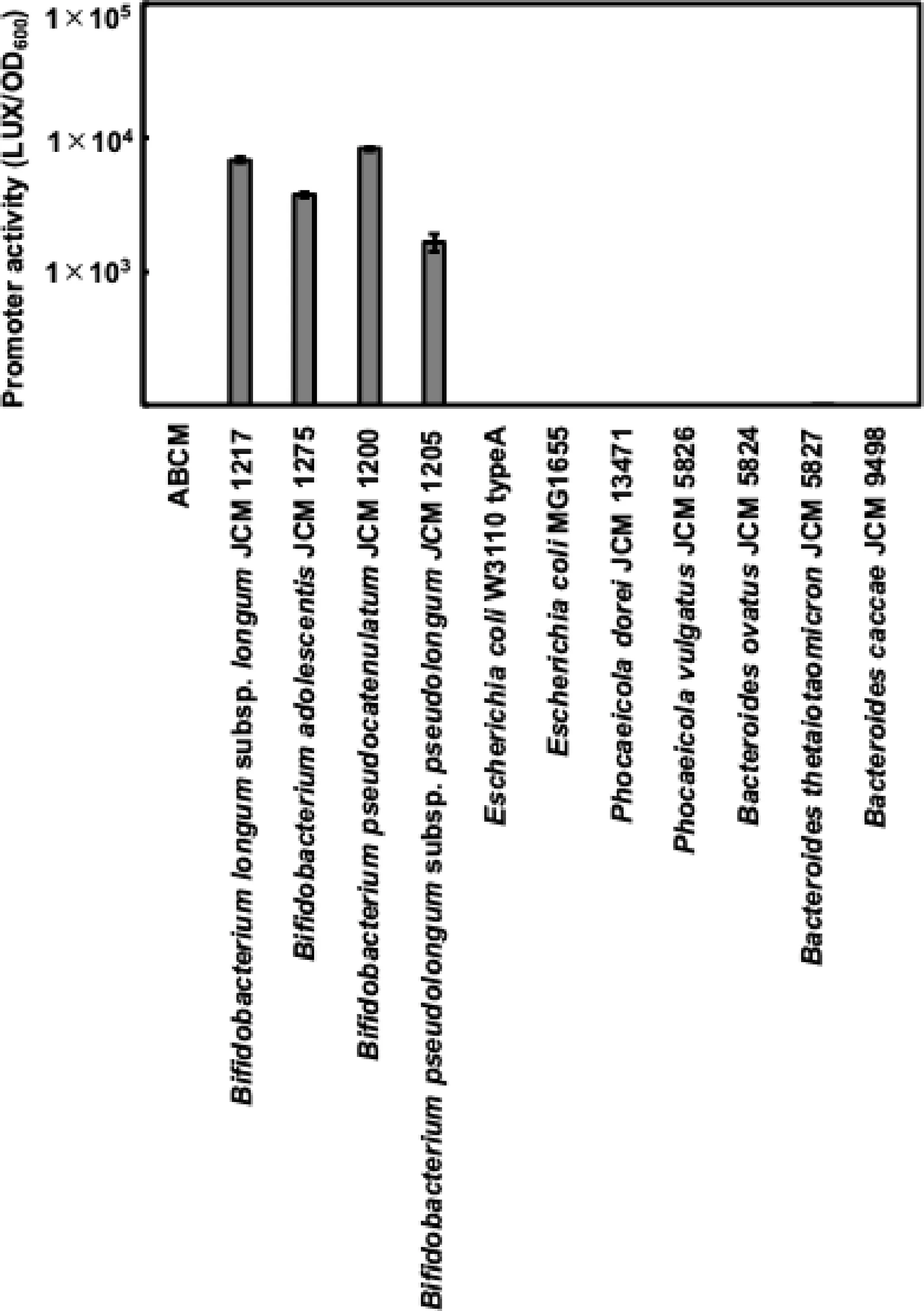
**Genus-specific induction of the** ***uhpT*** **promoter by** ***Bifidobacterium*** **species.** Screening of *uhpT* promoter activation in response to culture supernatants from various bacteria. The reporter strain, *Escherichia coli* BW25113 harbouring pLUX-uhpT, was exposed to supernatants from non-dominant constituents (four *Bifidobacterium* species and two *Escherichia coli* strains) and dominant constituents (five *Bacteroidota* species) (Table S2). Normalized bioluminescence (LUX/OD_600_) was monitored after 2.5 h of growth. All tested *Bifidobacterium* species induced significant promoter activity, whereas no induction was observed with other genera, indicating a genus-specific signaling mechanism. Data represent the means ± SD from three independent experiments. Statistical significance between the Parent and each mutant was evaluated using a two-tailed Student’s t-test (*p* < 0.001 for all *Bifidobacterium* species compared to ABCM).

The Uhp-mediated response is a specialized trait triggered by the genus *Bifidobacterium*. To determine whether the induction of the *uhpT* promoter is a specific response to *Bifidobacterium* or a general reaction to gut microbial metabolites, we screened culture supernatants from a phylogenetically diverse panel of intestinal bacteria. This panel included non-dominant constituents (four *Bifidobacterium* species and two *Escherichia coli* strains) and dominant constituents (five *Bacteroidota* species) (Table S2). Luciferase reporter assays revealed that all tested *Bifidobacterium* species significantly induced *uhpT* promoter activity, although the magnitude varied by species (Fig. 4). Among these strains, *Bifidobacterium pseudocatenulatum* JCM 1200 exhibited particularly robust induction (approximately 8,000 units) (Fig. 4). In striking contrast, no detectable induction was observed in response to supernatants from *Escherichia coli* (W3110 and MG1655) or any of the five *Bacteroidota* species (*Phocaeicola dorei*,* Bacteroides ovatus*,* Phocaeicola vulgatus*,* Bacteroides thetaiotaomicron*, and *Bacteroides caccae*), despite their dominance in the human gut. These results demonstrate that the Uhp-inducing factor—identified as G6P or its analogs—is not a universal byproduct of bacterial metabolism but is specifically secreted by the genus *Bifidobacterium*. The lack of response to *Bacteroides* secretions is particularly noteworthy, as these bacteria are major primary degraders of complex polysaccharides but do not appear to release hexose phosphates into the extracellular milieu in a manner perceivable by *Escherichia coli*. This genus-specific perception reinforces the model that the Uhp system serves as a specialized sensory channel for *Escherichia coli* to monitor the presence and metabolic footprint of *Bifidobacterium* within the competitive gut ecosystem.

### Identification of the Inducing Factor via a “Kinetic Trap” Approach

**Fig. 5:**
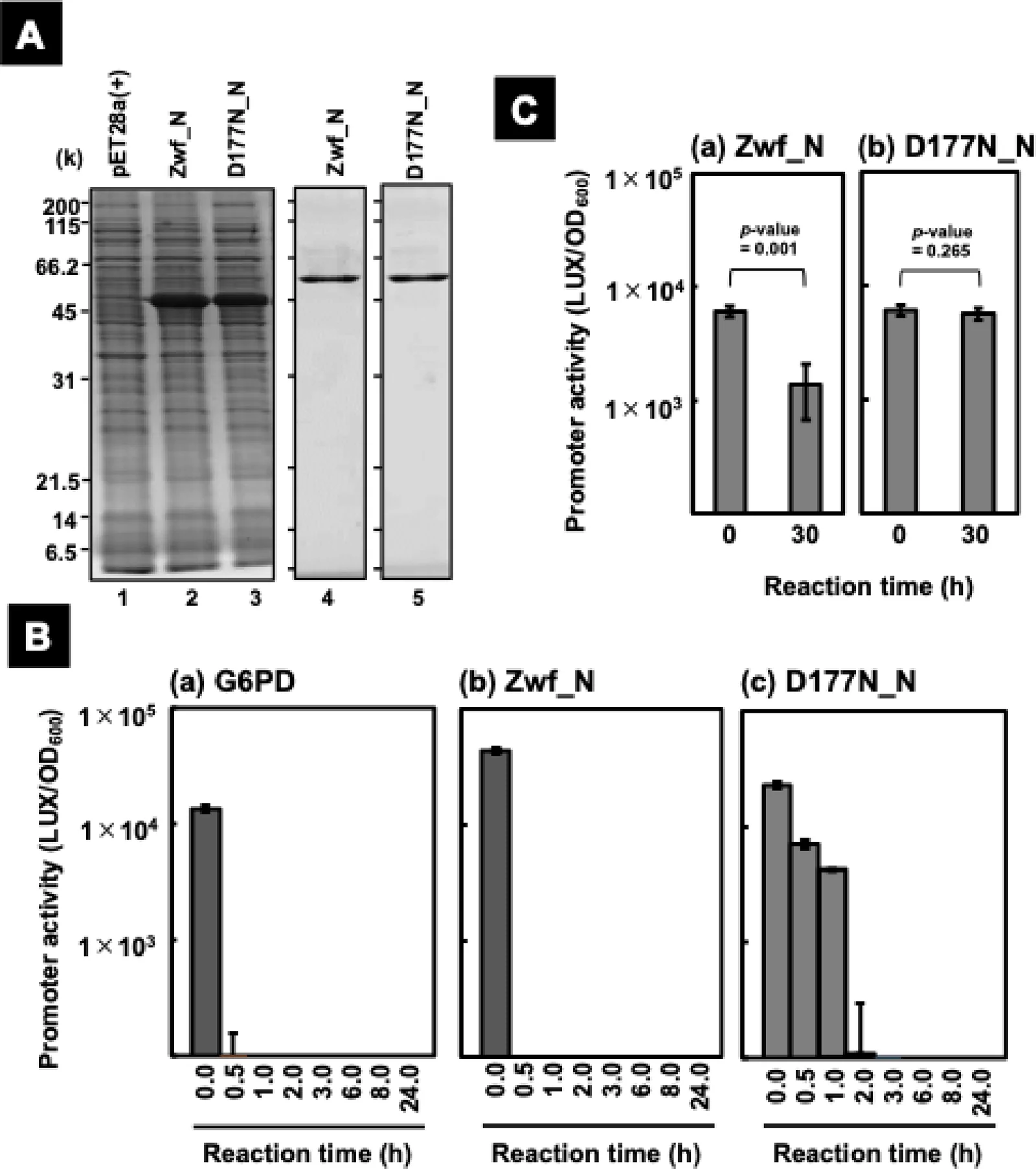
**Identification of the inducing factor in** ***Bifidobacterium*** **supernatant using the “Kinetic Trap” approach.** [A] SDS-PAGE analysis of recombinant protein expression and purification. Curde extracts were prepared from *Escherichia coli* BL21(DE3) harbouring pET28(+) (lane 1), pET28(+)_zwf_N (lane 2), and pET28(+)_zwf_D177N_N (lane 3). Purified His-tagged wild-type Zwf (Zwf_N; lane 4) and the D177N mutant (Zwf_D177N_N; lane 5) were obtained via Ni-NTA affinity chromatography. The gel images were cropped and adjusted for contrast using ImageJ. The original, full-length CBB-stained gel images of both the crude extracts and purified proteins are provided in Figure S2 in the Supplementary Information. [B] Verification of enzymatic activity in ABCM. The *uhpT*-inducing capacity of 350 µM G6P was monitored over time following treatment with commercial G6PD (a), Zwf_N (b), or Zwf_D177N_N (c). The slower inactivation rate of the D177N mutant compared to Zwf_N is consistent with its reduced catalytic velocity. [C] Inactivation of the inducing factors in *Bifidobacterium* culture supernatant by purified Zwf proteins. Supernatants were incubated with 10 µg/mL of Zwf_N or Zwf_D177N_N for 30 min before assessing residual *uhpT* promoter activity. Data represent the means ± SD from three independent experiments. Statistical significance between 0 and 30 min was evaluated using a two-tailed Student’s *t*-test (*p*-values are indicated above the brackets). The rapid inactivation by Zwf_N, but not the D177N mutant, identifies G6P or a related hexose phosphate as the primary signaling molecule.

The primary inducing factor in *Bifidobacterium* supernatant is sensitive to G6P-specific oxidation. To determine whether the inducing factor is G6P, we employed an enzymatic inactivation assay using G6P dehydrogenase (Zwf) from *Leuconostoc mesenteroides*^26^. We utilized two recombinant forms: the wild-type enzyme (Zwf_WT) and a D177N mutant (Zwf_D177N) (Fig. 5A), which exhibits a significantly reduced catalytic rate and functions as a “kinetic trap” by maintaining the substrate in a bound state^26^. In this trapped state, the enzyme effectively sequesters G6P molecules within its active site, thereby physically hindering their interaction with the membrane-bound UhpC receptor and consequently suppressing the induction of the *uhpT* promoter. Prior to testing the culture supernatant, we verified the activity of the purified enzymes in ABCM medium supplemented with 350 µM G6P and 0.86 mM NADP^+^. In this control assay, Zwf_WT and commercial G6P dehydrogenase (G6PD) completely abolished *uhpT* promoter activity within 1 h, confirming the near-complete conversion of the G6P substrate (Fig. 5B). In contrast, the D177N mutant required 3 h to achieve complete inactivation, consistent with its reduced catalytic velocity and confirming its functionality as a kinetic control in this medium (Fig. 5B). We then treated the *Bifidobacterium*-conditioned medium (BLCM) with 10 µg/mL of either Zwf_WT or Zwf_D177N for 30 min (Fig. 5C). Treatment with Zwf_WT resulted in an approximately 10-fold reduction in *uhpT* promoter activity compared to the untreated control (*p* = 0.001, Fig. 5C). Conversely, treatment with the D177N mutant caused no significant decrease in inducing activity within the same incubation period (*p* = 0.265, Fig. 5C). This rapid inactivation by the wild-type enzyme—but not by the slow-acting mutant—provides biochemical evidence that the primary inducing factor secreted by *Bifidobacterium* is G6P or a structurally related hexose phosphate that serves as a specific substrate for Zwf. These results identify G6P as the key molecular signal mediating the interaction between *Bifidobacterium* and the *Escherichia coli* Uhp two-component system.

### Induction of the *uhpT* Promoter by Supernatants of *Bifidobacterium* Grown on Alternative Carbon Sources

**Fig. 6:**
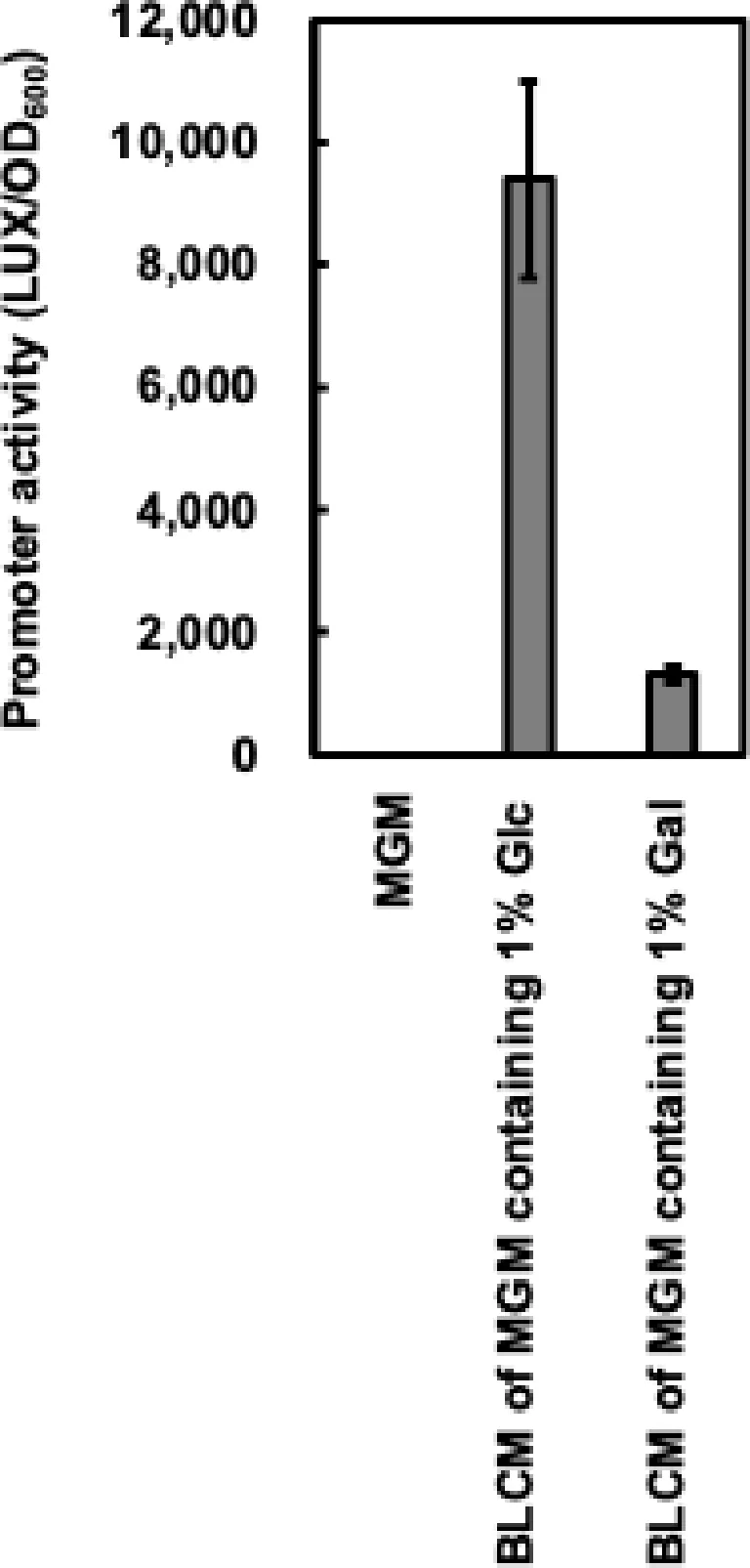
**Induction of the** ***uhpT*** **promoter by** ***Bifidobacterium longum*** **culture supernatants grown on alternative carbon sources.** *Bifidobacterium longum* subsp. *longum* JCM 1217 was cultured in Modified Garche’s Medium (MGM) supplemented with 1.0% (w/v) glucose or galactose as the sole carbon source. The resulting culture supernatants (BLCM) were used to stimulate the reporter strain, *Escherichia coli* BW25113 harboring pLUX-uhpT. Normalized bioluminescence (LUX/OD_600_) was monitored after 2.5 h of growth. Data represent the means ± SD from three independent experiments.

*Bifidobacterium* species are proficient at degrading HMOs and utilizing the liberated sugars for nutrition^18–21^. To determine whether the secretion of the G6P-like signaling molecule occurs during the metabolism of diverse carbohydrates, we examined the response of *Escherichia coli* to supernatants from *Bifidobacterium longum* subsp. *longum* JCM 1217 grown on various monosaccharide constituents of HMOs, including glucose, galactose, N-acetylglucosamine (GlcNAc), fucose, and N-acetylneuraminic acid (sialic acid). *Bifidobacterium* was cultured in Modified Garche’s Medium supplemented with 1.0% (w/v) of each sugar as the sole carbon source. While robust growth was confirmed in the presence of glucose or galactose, no growth was observed with GlcNAc, fucose, or sialic acid under these specific experimental conditions. We then prepared culture supernatants (BLCM) from the glucose- and galactose-grown cultures to assess *uhpT* promoter activity. The results showed significant induction with the glucose-derived supernatant. Notably, the galactose-derived supernatant also triggered induction, albeit at approximately 14% of the magnitude observed with glucose (Fig. 6). These findings demonstrate that the secretion of the molecular signal—identified as G6P—is not an exclusive byproduct of glucose excess but is a consistent phenomenon that occurs during the metabolism of other physiologically relevant sugars, such as galactose.

## Discussion

The present study demonstrates that *Escherichia coli* monitors the metabolic activity of *Bifidobacterium* through the Uhp two-component system (Figs. 2 and 3). Our biochemical “kinetic trap” analysis using Zwf_D177N provides strong biochemical evidence that the inducing factor is G6P or a structurally related hexose phosphate secreted by *Bifidobacterium* (Fig. 5). This induction is remarkably genus-specific; while all tested *Bifidobacterium* species triggered the Uhp system, no such response was observed with numerically dominant members of the *Bacteroidota*, such as *Phocaeicola* or *Bacteroides* (Fig. 4). Although these dominant taxa possess extensive machinery for complex carbohydrate degradation, they do not appear to release hexose phosphates into the extracellular environment in a manner detectable by the Uhp system. This suggests that the Uhp system in *Escherichia coli* appears to be tuned to sense the metabolic footprint of *Bifidobacterium* within the gut community.

The physiological relevance of this signaling mechanism is further supported by its occurrence during the metabolism of diverse carbon sources. Our results using Modified Garche’s Medium show that the G6P-mediated signal is secreted not only during growth on glucose but also during the utilization of galactose (Fig. 6). While induction with galactose was approximately 14% of that observed with glucose (Fig. 6), its presence across different sugars indicates that G6P release is a consistent byproduct of bifidobacterial carbohydrate metabolism rather than a specific response to glucose excess. Since G6P serves as a central hub in central carbon metabolism, its extracellular presence provides a reliable proxy for the metabolic activity of neighboring *Bifidobacterium*. While the present study utilized *Bifidobacterium longum* subsp. *longum* JCM 1217—a representative adult-derived strain—a similar signaling mechanism is anticipated in infant-type bifidobacteria, such as *B. longum* subsp. *infantis*, which possess more extensive machinery for HMO utilization. Given that G6P serves as a universal hub in bifidobacterial carbohydrate metabolism, these infant-associated species are expected to secrete similar hexose phosphate signals during the degradation of complex glycans, thereby facilitating metabolic synchronization with *Escherichia coli* through the Uhp system.

This inter-species communication is likely governed by strict biophysical and spatial constraints. G6P is a highly transient molecule, subject to rapid hydrolysis by microbial and host phosphatases. Furthermore, its diffusion in structured environments is physically constrained, with a self-diffusion coefficient (*D*) of G6P in hydrogel models at 37 °C is estimated at (4.6 ± 0.1) × 10^− 10^ m^2^ s^− 1^, representing a significant reduction compared to bulk aqueous solution^27^. These constraints imply that *Escherichia coli* must maintain close spatial proximity to perceive the signal, a model supported by recent spatial metagenomic insights revealing that the gut microbiome is organized into micron-scale spatial hubs (~ 40 μm in diameter) where specific taxa, including *Bifidobacterium* and members of the *Bacteroidota*, repeatedly co-localize^28^. In this context, the Uhp system serves as a sensory channel for metabolic eavesdropping (Fig. 1), allowing *Escherichia coli* to perceive the proximity of a metabolic partner.

Beyond simple nutrient acquisition, this interaction represents a sophisticated form of information sharing. The observation that *idnT* induction remains intact even in a Δ*uhpT* background (Fig. 2C) suggests that *Bifidobacterium* secretes a metabolic cocktail of signals, including both hexose phosphates and gluconates. By sensing these diverse secretions through independent systems (the Uhp two-component system and IdnR/GntR), *Escherichia coli* can synchronize its gene expression with the activity of partners, such as the liberated sugars resulting from species-specific HMO degradation by specific bifidobacteria like *Bifidobacterium bifidum*^18–21^.

We propose that these specific molecular links constitute a “non-dominant network” that facilitates survival within the highly competitive gut ecosystem. While dominant phyla, such as the *Bacteroidota* and *Bacillota*, engage in broad competitive strategies for dietary fibers, non-dominant taxa like *Escherichia coli* (*Pseudomonadota*) and *Bifidobacterium* (*Actinomycetota*) may rely on these precise inter-species sensory channels to occupy specialized niches. In this model, the Uhp two-component system serves as a critical component of a regulatory layer that enables low-abundance species to coordinate their metabolism and maintain stability in the shadow of the dominant majority. Such molecular interactions among non-dominant members likely contribute significantly to the overall diversity and resilience of the human microbiome, highlighting the necessity of looking beyond numerically dominant species to understand the true functional complexity of microbial ecology.

## Methods

### Bacterial strains, plasmids, oligonucleotides, and culture conditions

The bacterial strains, plasmids, and oligonucleotides used in this study are listed in Table S2. To generate in-frame deletions, the kanamycin (Km) resistance cassette was removed from *Escherichia coli* K-12 JW5702, JW3641, JW3642, JW3643, and JW3644 using the recombinase system of pKD46^29^. *Escherichia coli*, *Bifidobacterium* species, *Bacteroides* species were cultured in ABCM (Aerobic-anaerobic Culture Medium) broth (Eiken Chemical, Taito, Tokyo, Japan). The composition of the ABCM broth is as follows: 2.0% peptone, 0.5% yeast extract, 0.5% soluble starch, 0.3% beef extract, 0.3% soy peptone, 0.3% glucose, 0.25% K_2_HPO_4_, 0.2% vegetable extract, 0.2% NaCl, 0.03% L-cysteine HCl, 0.03% sodium thioglycolate, and 0.0005% hemin. To evaluate the utilization of specific carbon sources, Modified Garche’s Medium was employed. The Modified Garche’s Medium was composed of 2.0% Bacto peptone (BD Biosciences, Franklin Lakes, NJ, USA), 0.6% sodium acetate, 0.25% Na_2_HPO_4_·12H_2_O, 0.2% KH_2_PO_4_, 0.2% Bacto yeast extract (BD Biosciences), 0.045% L-cysteine HCl monohydrate, 0.012% MgSO_4_·12H_2_O, and 1% specific carbon source (e.g. glucose or galactose). For anaerobic cultivation, cultures were incubated at 37 °C in a CO_2_ atmosphere established by vacuum/refill cycle. *Bifidobacterium*-conditioned medium (BLCM) was prepared by harvesting the supernatant of a 15-h culture, adjusting the pH to 7.2, and filter-sterilizing it before diluting 1:4 with fresh ABCM. Antibiotics were added as required: kanamycin (50 µg/mL).

### Phenotype microarray (PM) analysis

Metabolic profiling of *Escherichia coli* W3110 was conducted using the OmniLog PM system (Biolog, Hayward, CA, USA). Cells were prepared in IF-0 inoculation fluid to a transmittance of 42% and distributed into PM1 plates. Substrate utilization was monitored every 15 min during a 96-h incubation at 37 °C via the colorimetric measurement of the reduction of a tetrazolium-based redox dye (Dye Mix A, Biolog).

### Plasmid construction and reporter assays

Lux-fusion reporter plasmids (e.g., pLUX-uhpT, pLUX-idnDOTR) were constructed by inserting approximately 500-bp promoter regions into the pLUX vector using the In-Fusion HD Cloning Kit (Clontech, Mountain View, CA, USA). For luciferase assays, *Escherichia coli* strains harboring these plasmids were grown for 2.5 h at 37 °C in ABCM or BLCM. Promoter activity was determined by measuring bioluminescence normalized to cell density (LUX/OD_600_) using an MTP-880 microplate reader (Corona Electric, Hitachinaka, Ibaraki, Japan).

### RNA isolation and expression analysis

Total RNA was extracted from *Escherichia coli* cells using the hot phenol method followed by treatment with Recombinant DNase I (Takara Bio, Kusatsu, Shiga, Japan). RNA integrity was verified by agarose gel electrophoresis. For RT-qPCR, cDNA was synthesized using the PrimeScript 1 st strand cDNA Synthesis Kit (Takara Bio). Quantitative PCR was performed on a 7500 Real-Time PCR System (Applied Biosystems, Foster City, CA, USA) using SYBR Green Master Mix, with *rrnH* as an internal control for normalization via the ΔCt method. For Northern blot analysis, digoxigenin (DIG)-labeled DNA probes were generated by PCR using specific primers (Table S2) and the DIG-dNTP mix (Roche, Mannheim, Germany). Denatured total RNA (10 µg) was separated on 1% formaldehyde-agarose gels and transferred to nylon membranes (Roche) via capillary transfer. After UV cross-linking, membranes were hybridized with DIG-labeled probes and detected using anti-DIG-AP antibody and CDP-Star chemiluminescent substrate (Roche).

### Protein purification and enzymatic assays

The *zwf* gene from *Leuconostoc mesenteroides* subsp. *mesenteroides* JCM 6124 and its D177N mutant (generated using QuikChange site-directed mutagenesis kit; Agilent Technologies, Santa Clara, CA, USA) were cloned into pET28a(+) vector. His-tagged proteins were overexpressed in *Escherichia coli* BL21(DE3) with 0.1 mM isopropyl-β-D-1-thiogalactopyranoside (IPTG) and purified using Ni-NTA agarose (Qiagen, Hilden, Germany) affinity chromatography. For the kinetic trap assay, purified Zwf proteins (10 µg/mL) were incubated with *Bifidobacterium*-conditioned medium (BLCM) or 350 µM G6P in ABCM containing 0.86 mM NADP^+^ at 25 °C. Residual inducing activity was then measured using the *uhpT-lux* reporter strain (*Escherichia coli* BW25113 harboring pLUX-uhpT) as described above.

## Supplementary Information

Below is the link to the electronic supplementary material.

Supplementary Material 1

## Data Availability

The datasets generated during and/or analyzed during the current study are available from the corresponding author on reasonable request. The *E. coli* strains and plasmids will be provided from NBRP of Japan.
