# Supplementary material for: The Escherichia coli uhp two-component system functions as a sensory channel for hexose phosphates secreted by Bifidobacterium
Source: Sci Rep. 2026 Aug 25;16:26318. doi: 10.1038/s41598-026-64276-3 (PMC13507172; doi:10.1038/s41598-026-64276-3)
Supplement: Supplementary file 1 [file 41598_2026_64276_MOESM1_ESM.pdf]

Supplementary Information

The *Escherichia coli* Uhp Two-Component System Functions as a Sensory Channel for Hexose Phosphates Secreted by *Bifidobacterium*

Tomotsugu Ohno, Masato Hoshino, Tsubasa Niino, Masanori Tamura, Miho Yoshimura, Kaneyoshi Yamamoto
Department of Frontier Bioscience and Research Institute of Micro-Nano Technology, Hosei University, Koganei, Tokyo 184-8584, Japan

Contents:

Figure S1. Full-length membrane image for the Northern blot presented in Figure 3A.
Figure S2. Full-length gel images for the SDS-PAGE presented in Figure 5A.
Table S1. The growth of W3110 typeA in PM1.
Table S2. Bacterial strains, plasmids, and oligonucleotides used in this study.
Reference

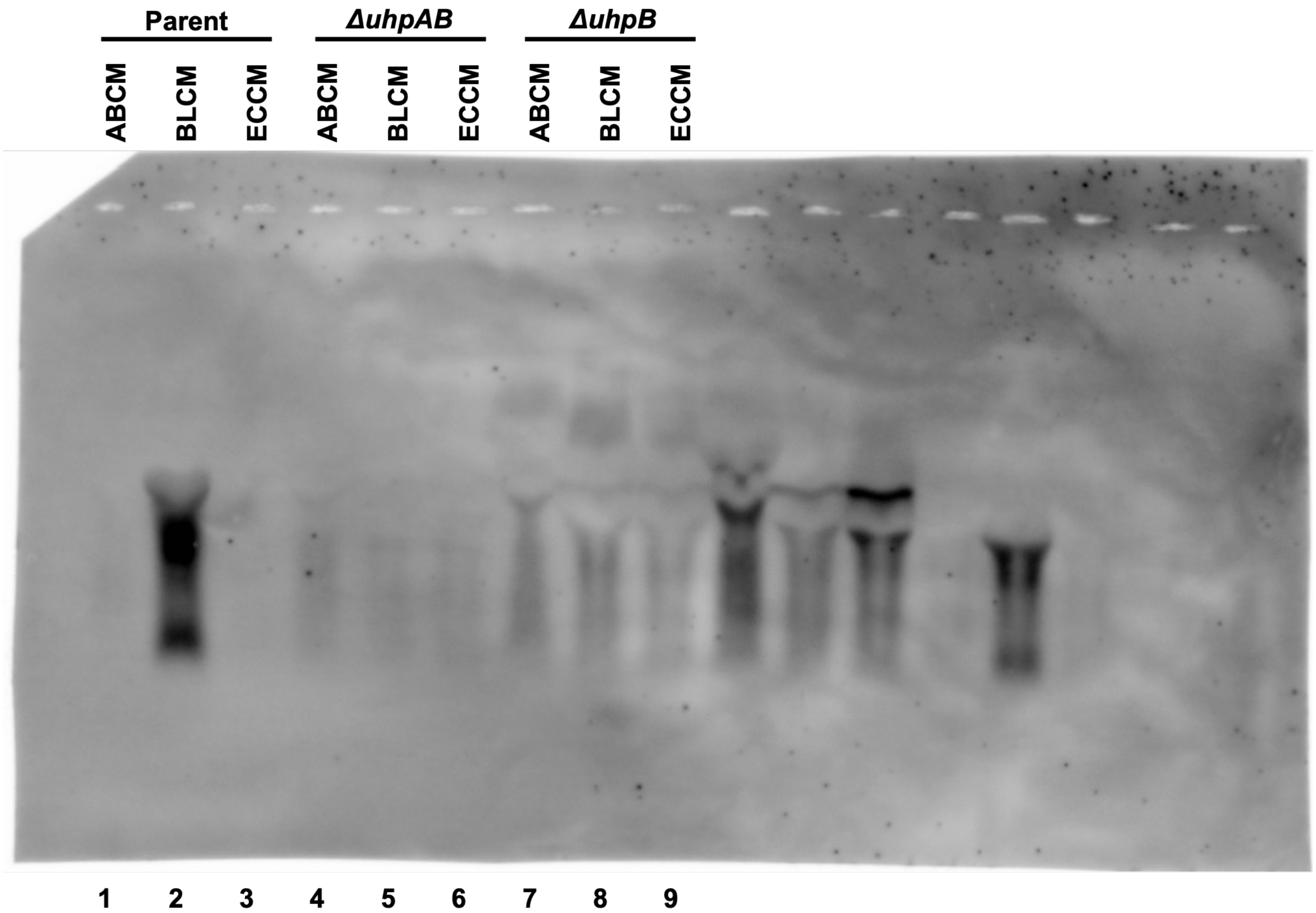

Figure S1. Full-length membrane image for the Northern blot presented in Figure 3A. The original, uncropped chemiluminescent image of the Northern blot is shown. Total RNA was isolated from *Escherichia coli* strains grown in ABCM (control), BLCM (*Bifidobacterium longum* subsp. *longum* JCM 1217 supernatant), or ECCM (*E. coli* supernatant). The membrane was hybridized with a DIG-labeled probe specific to the *uhpT* coding region. The sample loading order is as follows: Lanes 1–3: Parent (BW25113) grown in ABCM, BLCM, and ECCM, respectively. Lanes 4–6: *uhpAB* (BW27871) grown in ABCM, BLCM, and ECCM, respectively. Lanes 7–9: *uhpB* (JW3643Km) grown in ABCM, BLCM, and ECCM, respectively.

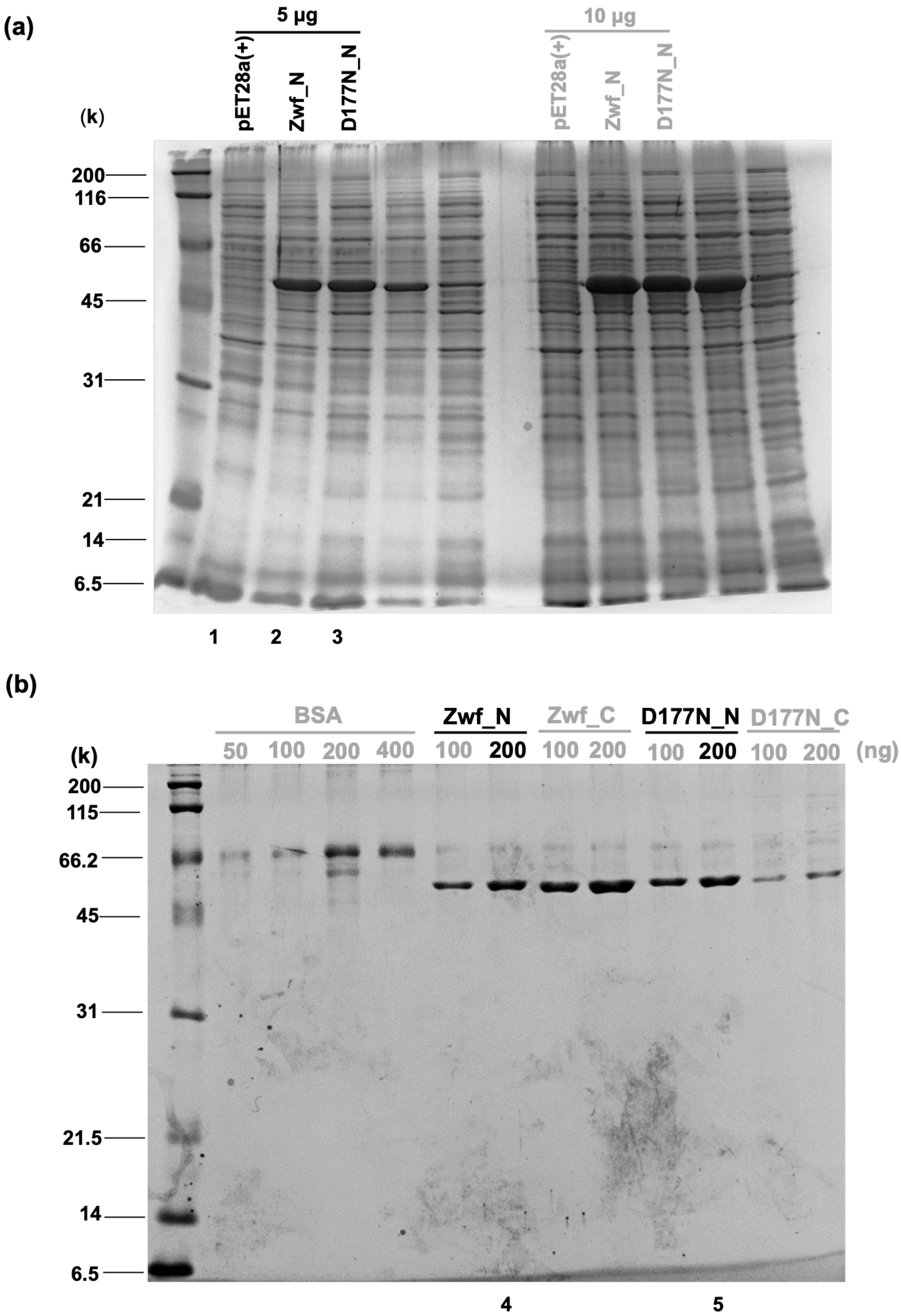

Figure S2. Full-length gel images for the SDS-PAGE presented in Figure 5A. The original, uncropped CBB-stained gel images of the recombinant protein analysis are shown. (a) Crude extracts prepared from *Escherichia coli* BL21(DE3) cells harboring various plasmids. The sample loading order is as follows: Lane 1: *E. coli* BL21(DE3) harboring pET28a(+) (empty vector control). Lane 2: *E. coli* BL21(DE3) harboring pET28a(+)_zwf_N. Lane 3: *E. coli* BL21(DE3) harboring pET28a(+)_zwf_D177N_N. For each sample, two different loading amounts (5 μg and 10 μg of total protein) are presented to confirm induction levels. (b)

Purified His-tagged proteins and bovine serum albumin (BSA) standards for quantification. The sample loading order corresponding to the representative lanes in Figure 5A is as follows: Lane 4: Purified wild-type Zwf (Zwf_N, 200 ng). Lane 5: Purified D177N mutant (D177N_N, 200 ng). BSA was loaded at 50, 100, 200, and 400 ng as protein concentration standards. (k) denotes the molecular weight marker in kilodaltons.

Table S1. The growth of W3110 typeA in PM1.

Carbon source	W3110 typeA	
	Experiment #1	Experiment #2
<C12 carbohydrate>		
D-Cellobiose	0	0
<C6 carbohydrate>		
α-D-Glucose	152	117
D-Glucose-6- Phosphate	228	201
D-Glucose-1- Phosphate	198	170
D-Mannose	141	120
D-Galactose	122	147
D-Fructose	160	121
D-Fructose-6- Phosphate	207	168
D-Sorbitol	207	180
Citric Acid	0	0
D-Galacturonic Acid	274	215
D-Gluconic Acid	221	201
D-Glucosaminic Acid	0	0
D-Glucuronic Acid	214	200
D-Mannitol	170	126
<C5 carbohydrate>		
L-Arabinose	131	113
D-Xylose	184	143
D-Ribose	188	169
Adonitol	0	0
<C4 carbohydrate>		
Acetoacetic Acid	0	0
Bromo Succinic Acid	0	0
D-Malic Acid	0	0
<C3 carbohydrate>		

1,2-Propanediol	0	0
<C2 carbohydrate>		
Acetic Acid	163	131
2-Aminoethanol	0	0
<Other>		
2-Deoxy Adenosine	227	192
Adenosine	223	180
D-Alanine	183	168
D-Aspartic Acid	0	0
D-Galactonic Acid-γ-Lactone	204	167
D-Melibiose	185	149
D-Psicose	0	0
D-Saccharic Acid	214	196
D-Serine	221	183
D-Threonine	0	0
D-Trehalose	148	159
D,L-Malic Acid	0	0
D,L-α-Glycerol- Phosphate	0	0
Dulcitol	0	0
Formic Acid	0	0
Fumaric Acid	0	0
Glucuronamide	0	0
Glycerol	115	99
Glycolic Acid	170	141
Glycyl-L- Aspartic Acid	183	141
Glycyl-L- Glutamic Acid	143	96
Glycyl-L- Proline	178	124
Glyoxylic Acid	110	61
Inosine	244	208
L-Alanine	186	148
L-Alanyl- Glycine	189	155

L-Asparagine	124	100
L-Aspartic Acid	0	0
L-Fucose	138	121
L-Galactonic Acid-γ-Lactone	242	140
L-Glutamic Acid	182	126
L-Glutamine	170	134
L-Lactic Acid	144	140
L-Lyxose	229	160
L-Malic Acid	0	0
L-Proline	204	161
L-Rhamnose	148	133
L-Serine	178	132
L-Threonine	0	0
Lactulose	226	156
m-Hydroxy Phenyl Acetic	0	0
M-Inositol	0	0
M-Tartaric Acid	0	0
Maltose	144	126
Maltotriose	198	160
Methyl Pyruvate	205	170
Mono Methyl Succinate	0	0
Mucic Acid	226	180
N-Acetyl-D- Glucosamine	173	109
N-Acetyl-β-D- Mannosamine	179	131
p-Hydroxy Phenyl Acetic	0	0
Phenylethyl- amine	0	0
Propionic Acid	172	131
Pyruvic Acid	236	181
Succinic Acid	90	86
Sucrose	0	0
Thymidine	226	193
Tricarballylic Acid	0	0
Tween 20	0	0
Tween 40	0	0
Tween 80	0	0

Tyramine	0	0
Uridine	255	220
α-D-Lactose D10 Lactulose	163	103
α-Hydroxy Butyric Acid	155	118
α-Hydroxy Glutaric Acid-γ- Lactone	0	0
α-Keto-Butyric Acid	166	138
α-Keto-Glutaric Acid	177	151
α-Methyl-D- Galactoside	209	184
β-Methyl-D- Glucoside	189	155

The arbitrary unit is represented after a 96-hour incubation period. The experiment was performed twice.

Table S2. Bacterial strains, plasmids, and oligonucleotides used in this study.

Name	Characterization	Reference
**Bacterial strains**		
Escherichia coli K-12 MG1655	F^-, λ^-, *ilvG*$^-$, *rfb*-50, *rph*-1	Hayashi et al., 2006
Escherichia coli K-12 W3110 typeA	complete σ set	Jishage and Ishihama, 1997
Escherichia coli BL21(DE3)	F^-, *ompT*, *hsdSB*(rB^- mB^-), *gal*(λ*cI*857, *ind*1, Sam7, *nin*5, *lacUV*5-T7gene1), *dcm*(DE3)	Novagen
Escherichia coli K-12 BW25113	F^-, DE(*araD*-*araB*)567, *lacZ*4787(del)::*rrnB*-3, LAM$^-$, *rph*-1, DE(*rhaD*-*rhaB*)568, *hsdR*514	Datsenko and Wanner, 2000
Escherichia coli K-12 JW5702	BW25113, *crp*::Km	Baba et al., 2006
Escherichia coli K-12 JW3641	BW25113, *uhpT*::Km	Baba et al., 2006
Escherichia coli K-12 JW3642	BW25113, *uhpC*::Km	Baba et al., 2006
Escherichia coli K-12 JW3643	BW25113, *uhpB*::Km	Baba et al., 2006
Escherichia coli K-12 JW3644	BW25113, *uhpA*::Km	Baba et al., 2006
Escherichia coli K-12 JW5702ΔKm	BW25113, Δ*crp*	This study
Escherichia coli K-12 JW3641ΔKm	BW25113, Δ*uhpT*	This study
Escherichia coli K-12 JW3642ΔKm	BW25113, Δ*uhpC*	This study
Escherichia coli K-12 JW3643ΔKm	BW25113, Δ*uhpB*	This study
Escherichia coli K-12 JW3644ΔKm	BW25113, Δ*uhpA*	This study
Escherichia coli K-12 BW27871	BW25113, Δ*uhpAB*	Oshima et al., 2002
Bifidobacterium longum subsp. *longum* JCM 1217	Isolation from intestine of adult	Sakata, et al., 2002
Bifidobacterium adolescentis JCM 1275	Isolation from intestine of adult	Reuter, 1963
Bifidobacterium pseudocatenulatum JCM 1200	Isolation from feces infant	Lauer and Kandler, 1983
Bifidobacterium pseudolongum subsp. *pseudolongum* JCM 1205	Isolation from swine infant	Lauer and Kandler, 1983
Phocaeicola dorei JCM 13471	Isolation from human feces	Bakir et al., 2006
Phocaeicola vulgatus JCM 5826	Isolation from human feces	Johnson, 1978
Bacteroides ovatus JCM 5824	Isolation from human feces	Lauer and Kandler, 1983
Bacteroides thetaiotaomicron JCM 5827	Isolation from human feces	Johnson, 1978
Bacteroides caccae JCM 9498	Isolation from human feces	Johnson et al., 1986

Leuconostoc mesenteroides subsp. *mesenteroides* JCM 6124	Isolation from fermentating olives	Björkroth et al., 2000
**Plasmids**		
pLUX	*kan*, *luxCDABE*, STOP codons, ribosome binding site	Yamanaka, et al., 2020
pLUX-cmtBA	pLUX, *cmtBA* promoter-*luxCDABE*	This study
pLUX-idnDOTR	pLUX, *idnDOTR* promoter-*luxCDABE*	This study
pLUX-uhpT	pLUX, *uhpT* promoter-*luxCDABE*	This study
pET28a(+)	*kan*, T7 promoter expression plasmid	Novagen
pET28a(+)_zwf_N	pET28a(+), *zwf* gene expression	This study
pET28a(+)_zwf_D177N_N	pET28a(+), *zwf* gene (D177N mutantion) expression	This study
**Oligonucleotides**		
pLUX_R	5'-GGCAGGTAAACACTATTATCACC-3'	Yamanaka, et al., 2020
cmtB_LUX_F	5'-TCGTCTTCACCTCGAAAATTATCCTTATTTTATTTTTAGG-3' (For pLUX-cmtAcloning)	This study
cmtB_LUX_R	5'-ACTAACTAGAGGATCGAAAGCGGTTACTGCACGCGTTGCT-3' (For pLUX-cmtAcloning)	This study
idnT_LUX_F	5'-TCGTCTTCACCTCGAAAATTCCTCACTGGTCAGGTAGACA-3' (For pLUX-idnT cloning)	This study
idnT_LUX_R	5'-ACTAACTAGAGGATCAAGACTGTATGAAGCATCATTTAAG-3' (For pLUX-idnT cloning)	This study
UHPT_LR_F1	5'-TCGTCTTCACCTCGACTTTTTGAACGCCCAGACACCGCGC-3' (For pLUX-uhpT cloning)	This study
UHPT_LR_R1	5'-ACTAACTAGAGGATCAGCCAGCATGGGTTACTCCTGAAAT-3' (For pLUX-uhpT cloning)	This study
T7 Promoter	5'-TAATACGACTCACTATAGGG-3'	Novagen
T7 Terminator	5'-CTAGTTATTGCTCAGCGG-3'	Novagen
zwf_ET28_N_F	5'-CGCGCGGCAGCCATATGGTTTCAGAAATCAAAACGTTGGT-3' (For pET28a(+)_zwf_N cloning)	This study
zwf_ET28_N_R	5'-GTCATGCTAGCCATATTATCCTTTAAATACCCAAGCATCG-3' (For pET28a(+)_zwf_N cloning)	This study
Zwf_D177N_F	5'-CAACTGTTCCGTATTAACCACTATCTTGGAAAA-3' (For pET28a(+)_zwf_D177N_N cloning)	This study
Zwf_D177N_R	5'-TTTTCCAAGATAGTGGTTAATACGGAACAGTTG-3' (For pET28a(+)_zwf_D177N_N cloning)	This study
uhpT_O_NF	5'-GATCTGCCACTTGGTCATAT-3' (For northern blotting of *uhpT* mRNA)	This study
uhpT_O_NR	5'-ATGCTGGCTTTCTTAAACCA-3' (For northern blotting of *uhpT* mRNA)	This study

16s-982	5'-,CGATGCAACGCGAAGAACCT-3' (For RT-qPCR of *rrn*)	Kailasan Vanaja et al., 2009
16s-1143	5'-,GCCGGACCGCTGGCAACAAA-3' (For RT-qPCR of *rrn*)	Kailasan Vanaja et al., 2009
actP_RT_F	5'-AGGTGTCGACATCATGAAGC-3' (For RT-qPCR of *actP* gene)	This study
actP_RT_R	5'-TAGCCCATAAACCCGGTGGC-3' (For RT-qPCR of *actP* gene)	This study
agaC_RT_F	5'-TGGTCTTGGCCTGCCGTTTA-3' (For RT-qPCR of *agaC* gene)	This study
agaC_RT_R	5'-AAAGCAATCACCGCATACGC-3' (For RT-qPCR of *agaC* gene)	This study
agaW_RT_F	5'-ACGGCCTAACCCATATGCAC-3' (For RT-qPCR of *agaW* gene)	This study
agaW_RT_R	5'-ACGCCGTGCCGACGATAGTA-3' (For RT-qPCR of *agaW* gene)	This study
ansP_RT_F	5'-TGTTGATTCAGGGCGTAGTG-3' (For RT-qPCR of *ansP* gene)	This study
ansP_RT_R	5'-ACGCGCTCCACGGCAATAAC-3' (For RT-qPCR of *ansP* gene)	This study
araE_RT_F	5'-AAAGGGGCGTCATATTGAGG-3' (For RT-qPCR of *araE* gene)	This study
araE_RT_R	5'-GCCTGCAACAACATACCGAG-3' (For RT-qPCR of *araE* gene)	This study
araF_RT_F	5'-GCTGGTGATGATGGCGGCGA-3' (For RT-qPCR of *araF* gene)	This study
araF_RT_R	5'-AATCCGGCCGCTTTCAGCGC-3' (For RT-qPCR of *araF* gene)	This study
araJ_RT_F	5'-AGTCAGGAATTTAGCTGGCG-3' (For RT-qPCR of *araJ* gene)	This study
araJ_RT_R	5'-AAACATCGTGGCGGCGAAAA-3' (For RT-qPCR of *araJ* gene)	This study
ascF_RT_F	5'-CCGGTCACCGCGGTGAAATA-3' (For RT-qPCR of *ascF* gene)	This study
ascF_RT_R	5'-AATCGGGCCAATCAGCAGGA-3' (For RT-qPCR of *ascF* gene)	This study
bglF_RT_F	5'-TTGCCGCCGGTTATCTCTGG-3' (For RT-qPCR of *bglF* gene)	This study
bglF_RT_R	5'-TAATGGCGGGCATTAACAGC-3' (For RT-qPCR of *bglF* gene)	This study
citT_RT_F	5'-CATGTTTGTCACCGGTGCGG-3' (For RT-qPCR of *citT* gene)	This study
citT_RT_R	5'-TCACTGTGTGTGATTTCCGG-3' (For RT-qPCR of *citT* gene)	This study
cmtA_RT_F	5'-CCGCTGGGAATGCAACAGGC-3' (For RT-qPCR of *cmtA* gene)	This study
cmtA_RT_R	5'-ATACAGTTCGTGGATCCCAC-3' (For RT-qPCR of *cmtA* gene)	This study
cycA_RT_F	5'-AAGCGTCATTCGCGCATTTG-3' (For RT-qPCR of *cycA* gene)	This study
cycA_RT_R	5'-TCGGAATGGAGTTAATCGCG-3' (For RT-qPCR of *cycA* gene)	This study
cyuP_RT_F	5'-ACCGGCATGGTGCTGACCAA-3' (For RT-qPCR of *cyuP* gene)	This study

cyuP_RT_R	5'-CATCGGACTTAACGTCTGGA-3' (For RT-qPCR of *cyuP* gene)	This study
dhaM_RT_F	5'-CGGCAAATGCGTCACACCAG-3' (For RT-qPCR of *dhaM* gene)	This study
dhaM_RT_R	5'-GGACGCAGAGTAGGTGGAGC-3' (For RT-qPCR of *dhaM* gene)	This study
fruB_RT_F	5'-TGCTGACACTGGATATCGTC-3' (For RT-qPCR of *fruB* gene)	This study
fruB_RT_R	5'-CGCTACGCAGATTGCCTTCG-3' (For RT-qPCR of *fruB* gene)	This study
fucP_RT_F	5'-TATCGCGGTTGTCTTTGGGC-3' (For RT-qPCR of *fucP* gene)	This study
fucP_RT_R	5'-ATGATCAGCAGGGCGACCAG-3' (For RT-qPCR of *fucP* gene)	This study
gadC_RT_F	5'-CTGATGGTGGCGGCAATCTG-3' (For RT-qPCR of *gadC* gene)	This study
gadC_RT_R	5'-ACCCAGCAACAGCAGTGCGG-3' (For RT-qPCR of *gadC* gene)	This study
galP_RT_F	5'-TTGTTGATGCCGAACGCGTG-3' (For RT-qPCR of *galP* gene)	This study
galP_RT_R	5'-ATTGCTGCATTACCTGCAAC-3' (For RT-qPCR of *galP* gene)	This study
glcA_RT_F	5'-TACTTCGGCGCTGGTGAGTA-3' (For RT-qPCR of *glcA* gene)	This study
glcA_RT_R	5'-GACCACGCTCGAATGATTTG-3' (For RT-qPCR of *glcA* gene)	This study
glnH_RT_F	5'-GGCCTGTTAGTGATGGTGAA-3' (For RT-qPCR of *glnH* gene)	This study
glnH_RT_R	5'-GGTGCCCAGTTCCATATAGG-3' (For RT-qPCR of *glnH* gene)	This study
glpF_RT_F	5'-TTTCTGTGCTGCGGCTTTAG-3' (For RT-qPCR of *glpF* gene)	This study
glpF_RT_R	5'-GCGGTAATCACCATCTCAAC-3' (For RT-qPCR of *glpF* gene)	This study
glpT_RT_F	5'-CTTTCTGCGCCATTCTGGTG-3' (For RT-qPCR of *glpT* gene)	This study
glpT_RT_R	5'-GTTTGTTCGGCAGTACGTAC-3' (For RT-qPCR of *glpT* gene)	This study
gltI_RT_F	5'-CAAACCGGACTTGCAGGTAA-3' (For RT-qPCR of *gltI* gene)	This study
gltI_RT_R	5'-GGCTTTGTCTTTCAGGTTGG-3' (For RT-qPCR of *gltI* gene)	This study
gltP_RT_F	5'-CATCGTGAACCGCTGGTGAC-3' (For RT-qPCR of *gltP* gene)	This study
gltP_RT_R	5'-GGCGAAATGCACCAGCAGCA-3' (For RT-qPCR of *gltP* gene)	This study
gltS_RT_F	5'-GTGCAACGTTCGGTCTGGTG-3' (For RT-qPCR of *gltS* gene)	This study
gltS_RT_R	5'-CAAAAGCAGTGCCAGCCAAA-3' (For RT-qPCR of *gltS* gene)	This study
gntT_RT_F	5'-CGGTACTATTCTGGCAATCC-3' (For RT-qPCR of *gntT* gene)	This study
gntT_RT_R	5'-GCCATCAGTACTACCGGCAC-3' (For RT-qPCR of *gntT* gene)	This study

gntU_RT_F	5'-ATTATTGCCGGGCCGCTGTG-3' (For RT-qPCR of *gntU* gene)	This study
gntU_RT_R	5'-CACAAAACGCGCGGCAATGG-3' (For RT-qPCR of *gntU* gene)	This study
idnT_RT_F	5'-CGACAGTTATTGTCGCAGGA-3' (For RT-qPCR of *idnT* gene)	This study
idnT_RT_R	5'-TTTCACAAACGGCGGCGATA-3' (For RT-qPCR of *idnT* gene)	This study
kgtP_RT_F	5'-ACGCTGCACTCAGAGAGTGG-3' (For RT-qPCR of *kgtP* gene)	This study
kgtP_RT_R	5'-GAACCATGATGAATGCACGG-3' (For RT-qPCR of *kgtP* gene)	This study
lacY_RT_F	5'-ACTCATCCTCGCCGTTTTAC-3' (For RT-qPCR of *lacY* gene)	This study
lacY_RT_R	5'-TCGTAGGTGCAGGAAACGCC-3' (For RT-qPCR of *lacY* gene)	This study
lldP_RT_F	5'-TGCCGGACATTATCTCTTCG-3' (For RT-qPCR of *lldP* gene)	This study
lldP_RT_R	5'-GGAACAGGAACGGTGTCCAG-3' (For RT-qPCR of *lldP* gene)	This study
malE_RT_F	5'-AAAGCGAAAGGTAAGAGCGC-3' (For RT-qPCR of *malE* gene)	This study
malE_RT_R	5'-AATCAGGTCAACCAGGAAGG-3' (For RT-qPCR of *malE* gene)	This study
malX_RT_F	5'-TGGTCTGCATCACATTCTGG-3' (For RT-qPCR of *malX* gene)	This study
malX_RT_R	5'-GCAGGCATTTTACCTTGCGA-3' (For RT-qPCR of *malX* gene)	This study
manY_RT_F	5'-AGCATTGGTGCAGGTATCGC-3' (For RT-qPCR of *manY* gene)	This study
manY_RT_R	5'-ACGCATTGCTTGCAGGAACA-3' (For RT-qPCR of *manY* gene)	This study
melB_RT_F	5'-GTTCACTCTGGTACTGATCG-3' (For RT-qPCR of *melB* gene)	This study
melB_RT_R	5'-AGAGCCATACCCAAGAGGCA-3' (For RT-qPCR of *melB* gene)	This study
mglB_RT_F	5'-GCTACGACAAAGCCTACTAC-3' (For RT-qPCR of *mglB* gene)	This study
mglB_RT_R	5'-AAGTGGTACGTGCTTCTGCA-3' (For RT-qPCR of *mglB* gene)	This study
mtlA_RT_F	5'-GCGTCTGATCCTCGCAGTCA-3' (For RT-qPCR of *mtlA* gene)	This study
mtlA_RT_R	5'-AAGGAGACAGCCATCGCCGC-3' (For RT-qPCR of *mtlA* gene)	This study
nagE_RT_F	5'-TCATGTTCCTTGCTCCGCTG-3' (For RT-qPCR of *nagE* gene)	This study
nagE_RT_R	5'-CCATCACCAGCAGCATCCAG-3' (For RT-qPCR of *nagE* gene)	This study
nanT_RT_F	5'-CTACTGCGCTGTGGTTCTGC-3' (For RT-qPCR of *nanT* gene)	This study
nanT_RT_R	5'-TCGGCCATGAGTAGAGGAAA-3' (For RT-qPCR of *nanT* gene)	This study
ptsG_RT_F	5'-CACCACATCTGGAACGTACC-3' (For RT-qPCR of *ptsG* gene)	This study

ptsG_RT_R	5'-GTGCCAGATAGCAATTGCGG-3' (For RT-qPCR of *ptsG* gene)	This study
ptsP_RT_F	5'-CTTAGTCGGTGTTGTCGTGC-3' (For RT-qPCR of *ptsP* gene)	This study
ptsP_RT_R	5'-AGCAGTACCGGCTCCGGATC-3' (For RT-qPCR of *ptsP* gene)	This study
putP_RT_F	5'-CAGTGTCGGTGGCTTTGGTG-3' (For RT-qPCR of *putP* gene)	This study
putP_RT_R	5'-ATGCTGTGGTGAGAATCCGC-3' (For RT-qPCR of *putP* gene)	This study
rbsB_RT_F	5'-ATCACTCTTGACCGCCAGGC-3' (For RT-qPCR of *rbsB* gene)	This study
rbsB_RT_R	5'-GAAGCCTTCGCCACGTTCAC-3' (For RT-qPCR of *rbsB* gene)	This study
rhaT_RT_F	5'-GTTGATTAGCACCGAAGGCG-3' (For RT-qPCR of *rhaT* gene)	This study
rhaT_RT_R	5'-CCGGCAGAGAAAATGCCGCA-3' (For RT-qPCR of *rhaT* gene)	This study
sdaC_RT_F	5'-CTGTACCTGATCCCGCAGTG-3' (For RT-qPCR of *sdaC* gene)	This study
sdaC_RT_R	5'-GCCGTACTCTTCACGCTTCG-3' (For RT-qPCR of *sdaC* gene)	This study
srlA_RT_F	5'-CTGTTTCCCGTTACCTACTG-3' (For RT-qPCR of *srlA* gene)	This study
srlA_RT_R	5'-CAAACAGTTCGCCAGGGTTG-3' (For RT-qPCR of *srlA* gene)	This study
sstT_RT_F	5'-GTCACGGTAACGAGACCACC-3' (For RT-qPCR of *sstT* gene)	This study
sstT_RT_R	5'-CAACCAGCACGACCAGCAGT-3' (For RT-qPCR of *sstT* gene)	This study
tdcC_RT_F	5'-ATCGACCAGGTTGACCTCGG-3' (For RT-qPCR of *tdcC* gene)	This study
tdcC_RT_R	5'-ACGTTCGGTGAAGTCGCGAC-3' (For RT-qPCR of *tdcC* gene)	This study
treB_RT_F	5'-ACGCTTATCTGCTCGGGCAG-3' (For RT-qPCR of *treB* gene)	This study
treB_RT_R	5'-CGGGTACCACCACCAGATAG-3' (For RT-qPCR of *treB* gene)	This study
ttdT_RT_F	5'-TTTCTTGACGGCGATGGCAC-3' (For RT-qPCR of *ttdT* gene)	This study
ttdT_RT_R	5'-TGATCGCCAGACTTCAGTAC-3' (For RT-qPCR of *ttdT* gene)	This study
ugpB_RT_F	5'-GAAACTGAAAGCCTCCGGCA-3' (For RT-qPCR of *ugpB* gene)	This study
ugpB_RT_R	5'-AGCATGGCGATGTGTTTCAC-3' (For RT-qPCR of *ugpB* gene)	This study
uhpT_RT_F	5'-TCGGCATGTTTATCTTCCCG-3' (For RT-qPCR of *uhpT* gene)	This study
uhpT_RT_R	5'-CAAAGATCTGCCACTTGGTC-3' (For RT-qPCR of *uhpT* gene)	This study
xylE_RT_F	5'-CCTCGGAATGTATCCCTGCA-3' (For RT-qPCR of *xylE* gene)	This study
xylE_RT_R	5'-GGCCATGATCCAGGGAGTGT-3' (For RT-qPCR of *xylE* gene)	This study

xylF_RT_F	5'-AAAGTCGGTGAACTGCAGGC-3' (For RT-qPCR of *xylF* gene)	This study
xylF_RT_R	5'-ATCAACCCATTGGTCACCAA-3' (For RT-qPCR of *xylF* gene)	This study

Reference

Baba, T. et al. Construction of *Escherichia coli* K-12 in-frame, single-gene knockout mutants: the Keio collection. *Mol. Syst. Biol.* **2**, 2006.0008 (2006).

Bakir, M. A., Sakamoto, M., Kitahara, M., Matsumoto, M. & Benno, Y. *Bacteroides dorei* sp. nov., isolated from human faeces. *Int. J. Syst. Evol. Microbiol.* **56**, 1639–1643 (2006).

Björkroth, K. J. et al. Characterization of *Leuconostoc gasicomitatum* sp. nov., associated with spoiled raw tomato-marinated broiler meat strips packaged under modified-atmosphere conditions. *Appl. Environ. Microbiol.* **66**, 3764–3772 (2000).

Datsenko, K. A. & Wanner, B. L. One-step inactivation of chromosomal genes in *Escherichia coli* K-12 using PCR products. *Proc. Natl. Acad. Sci. U. S. A.* **97**, 6640–6645 (2000).

Hayashi, K. et al. Highly accurate genome sequences of *Escherichia coli* K-12 strains MG1655 and W3110. *Mol. Syst. Biol.* **2**, 2006.0007 (2006).

Jishage, M. & Ishihama, A. Variation in RNA polymerase sigma subunit composition within different stocks of *Escherichia coli* W3110. *J. Bacteriol.* **179**, 959–963 (1997).

Johnson, J. L. Taxonomy of the *Bacteroides*. I. Deoxyribonucleic acid homologies among *Bacteroides fragilis* and other *Bacteroides* species. Int. *J. Syst. Bacteriol.* **28**, 245–256 (1978).

Kailasan Vanaja, S., Bergholz, T. M. & Whittam, T. S. Characterization of the *Escherichia coli* O157:H7 Sakai GadE regulon. *J. Bacteriol.* **191**, 1868–1877 (2009).

Lauer, E. & Kandler, O. DNA-DNA homology, murein types and enzyme patterns in the type strains of the genus *Bifidobacterium*. *Syst. Applre. Microbiol.* **4**, 42–64 (1983).

Oshima, T. et al. Transcriptome analysis of all two-component regulatory system mutants of *Escherichia coli* K-12. *Mol. Microbiol.* **46**, 281–291 (2002).

Reuter, G. Vergleichende Untersuchungen über die Bifidus-Flora im Säuglings- und Erwachsenenstuhl. *Zentralbl. Bakteriol.* **191**, 486–507 (1963).

Sakata, S. et al. *Bifidobacterium thermacidophilum* subsp. *thermacidophilum* gen. nov., sp. nov. and *Bifidobacterium thermacidophilum* subsp. *porcinum* subsp. nov., isolated from human faeces and sewage. *Int. J. Syst. Evol. Microbiol.* **52**, 1945–1951 (2002).

Yamanaka, Y., Watanabe, H., Yamauchi, E., Miyake, Y. & Yamamoto, K. Measurement of the promoter activity in *Escherichia coli* by using a luciferase reporter. *Bio-protocol* **10**, e3500 (2020).
